# Protective Effect of Two Yeast Based Feed Additives on Pigs Chronically Exposed to Deoxynivalenol and Zearalenone

**DOI:** 10.3390/toxins6123336

**Published:** 2014-12-12

**Authors:** Alexandra C. Weaver, M. Todd See, Sung Woo Kim

**Affiliations:** Department of Animal Science, North Carolina State University, Raleigh, NC 27695, USA; E-Mails: acchayto@ncsu.edu (A.C.W.); todd_see@ncsu.edu (M.T.S.)

**Keywords:** deoxynivalenol, feed additives, pigs, yeast, zearalenone

## Abstract

To evaluate the effects of the mycotoxins deoxynivalenol (DON) and zearalenone (ZEA) on pigs and the benefits of two mycotoxin mitigation strategies, gilts (*n* = 84, 9.1 ± 0.1 kg) were allotted to four treatments: CON (control); MT (4.8 mg/kg feed DON and 0.3 mg/kg feed ZEA); MT-YC (MT + 2 g/kg of yeast cell wall product); and MT-YF (MT + 2 g/kg of yeast fermentation product). After 42 days of feeding, pigs fed MT had reduced (*p* < 0.05) growth performance compared with pigs fed CON. Pigs fed MT-YF had greater (*p* < 0.05) average daily gain and tended to have greater (*p* = 0.080) average daily feed intake than MT, whereas pigs fed MT-YC did not differ from MT. Oxidative DNA damage increased (*p* < 0.05) in MT, whereas pigs fed MT-YF tended to have lower (*p* = 0.067) oxidative stress. Liver hydropic degeneration was increased (*p* < 0.05) in MT in contrast to CON and MT-YF, and tended to be greater (*p* = 0.079) than MT-YC. Collectively, feeding diets contaminated with mycotoxins significantly reduced growth performance and impacted pig health. The yeast additives had varied ability to reduce mycotoxin effects on pig growth and health, but may still play a beneficial role in reducing the overall impacts of a mycotoxin challenge on pigs.

## 1. Introduction

Mycotoxins are toxic secondary metabolites produced by certain species of molds that commonly contaminate agricultural crops [1]. The fungus *Fusarium graminearum* has the ability to produce several mycotoxins including deoxynivalenol (DON) and zearalenone (ZEA). For both mycotoxins, swine are one of the most sensitive species [1,2,3]. Deoxynivalenol can impact gut health, alter brain neurotransmitter concentrations, alter immunity, and cause organ damage. On the other hand, ZEA has a structure similar to estradiol-17β that allows binding to estrogen receptors which can result in embryonic death, smaller litters, and smaller offspring [2,4,5]. 

Although not feeding animals mycotoxin contaminated grains is the ideal way to reduce the harmful effects of mycotoxins, contaminated feed may be unavoidable. Thus, to reduce toxic effects within the animal, feed additives with mycotoxin mitigation properties can play an important role [6,7]. Products containing yeast materials have potential to adsorb mycotoxins due to the physical properties of the yeast cell wall, which has structures that allow for binding of mycotoxins [8,9,10,11]. Some yeast materials may also improve the health of pigs through their prebiotic properties, which in turn can protect gut health, benefit the immune system, and improve performance [12,13]. 

Deoxynivalenol and ZEA have been previously shown to be harmful mycotoxins for swine, further information is needed on how these mycotoxins impact pig organ health, immunity, and oxidative stress when these mycotoxins simultaneously contaminate feedstuffs. The objective of this study was to determine the effects of feeding corn naturally contaminated with DON and ZEA on pig performance and health status. Additionally, this study investigated the ability of two yeast based feed additives to help pigs to manage the mycotoxin challenge. 

## 2. Results

### 2.1. Growth Performance

The initial body weight (BW) of pigs did not differ among treatments, nor did BW on day 7 (Table 1). On day 14, 21, and 28, pigs fed MT had reduced (*p* < 0.05) BW in contrast to CON, and the other treatments did not differ from MT. On day 35, pigs fed MT had reduced (*p* < 0.001) BW compared with CON, and tended (*p* = 0.061) to have reduced BW from MT-YF but not different from MT-YC. A similar effect was observed on day 42, where pigs fed MT had reduced (*p* < 0.001) BW from CON, and tended (*p* = 0.084) to have reduced BW from MT-YF. 

Average daily gain (ADG) of pigs fed MT was affected by feeding of the mycotoxins (Table 1). During day 0–7 pigs fed MT tended (*p* = 0.089) to have reduced ADG from CON only. During days 7–28, the ADG of pigs fed MT was decreased (*p* < 0.01) from pigs fed CON, but was not different from MT-YC and MT-YF. On day 28–35, pigs fed MT continued to have reduced (*p* < 0.05) ADG from CON, whereas MT-YF tended (*p* = 0.068) to improve ADG. However, from day 35–42, there was only a tendency (*p* = 0.093) for MT to have reduced ADG in contrast to CON. Throughout the entire study, pigs fed MT had decreased (*p* < 0.01) ADG compared with pigs fed CON. The ADG of pigs fed MT-YC was not different from the ADG of pigs fed MT, whereas pigs fed MT-YF had increased (*p* < 0.05) ADG from pigs fed MT. 

**Table 1:** Growth performance of pigs consuming 4.8 mg/kg deoxynivalenol (DON) and 0.3 mg/kg zearalenone (ZEA) with or without yeast based feed additives.

Item	Treatment ^1^				SEM	Contrast *p* value: MT *vs.*		
	CON	MT	MT-YC	MT-YF		CON	MT-YC	MT-YF
Body weight, kg								
Day 0	9.0	9.0	9.2	9.2	0.13	0.956	0.709	0.803
7	10.0	9.6	9.8	10.1	0.15	0.553	0.718	0.432
14	12.6	11.0	11.3	11.6	0.23	0.042	0.660	0.398
21	15.3	12.5	13.4	13.8	0.32	0.003	0.280	0.150
28	19.4	15.3	16.6	16.9	0.47	<0.001	0.219	0.137
35	22.9	17.7	19.7	20.2	0.62	<0.001	0.141	0.061
42	27.2	21.1	23.2	23.6	0.78	<0.001	0.155	0.084
ADG, g/day ^2^								
Days 0–7	143	82	89	139	19	0.089	0.840	0.110
7–14	365	196	208	210	25	<0.001	0.736	0.683
4–21	396	223	307	308	41	0.005	0.149	0.141
21–28	587	398	450	445	29	<0.001	0.216	0.264
28–35	499	346	436	476	47	0.034	0.197	0.068
35–42	606	483	501	488	50	0.093	0.798	0.942
0–42	433	288	332	344	18	<0.001	0.111	0.045
ADFI, g/day ^2^								
Days 0–7	351	254	274	316	20	0.005	0.527	0.061
7–14	616	365	411	437	20	<0.001	0.183	0.043
14–21	826	472	546	538	38	<0.001	0.226	0.276
21–28	1,094	640	740	735	44	<0.001	0.148	0.171
28–35	1,224	739	851	933	57	<0.001	0.207	0.034
35–42	1,350	913	1,020	1,017	70	<0.001	0.283	0.298
0–42	910	564	640	662	35	<0.001	0.169	0.080
Gain: Feed, g/g								
Days 0–7	0.395	0.293	0.298	0.441	0.049	0.229	0.950	0.086
7–14	0.592	0.540	0.508	0.488	0.055	0.505	0.683	0.510
14–21	0.475	0.457	0.564	0.578	0.063	0.834	0.237	0.182
21–28	0.534	0.626	0.608	0.619	0.030	0.041	0.675	0.881
28–35	0.402	0.470	0.513	0.512	0.034	0.164	0.376	0.387
35–42	0.449	0.535	0.491	0.495	0.044	0.176	0.486	0.526
0–42	0.476	0.512	0.517	0.521	0.012	0.043	0.778	0.596

^1^ CON: control without significant DON or ZEA; MT: 4.8 mg/kg DON and 0.3 mg/kg ZEA. Composition of diets MT-YC: MT + 2 g/kg of a yeast cell wall product (Integral, Alltech Inc., Nicholasville, KY, USA); and MT-YF: MT + 2 g/kg of a yeast fermentation product (Original XPC, Diamond V, Cedar Rapids, IA, USA); ^2^ ADG: average daily gain; ADFI: average daily feed intake.

The average daily feed intake (ADFI) of pigs fed MT was decreased (*p* < 0.01) from pigs fed CON throughout the trial (Table 1). The ADFI of pigs fed MT-YC was not different from MT during this study. Pigs fed MT-YF tended to have increased (*p* = 0.061) ADFI during days 0–7, and had increased ADFI during days 7–14, compared with pigs fed MT. The ADFI of MT-YF was not different from MT during days 14–28, but again was increased (*p* < 0.05) for days 28–35. However, during days 35–42, pigs fed MT-YF did not differ in ADFI from pigs fed MT. Over the entire trial period, pigs fed MT-YF tended to have increased (*p* = 0.080) ADFI in contrast to pigs fed MT.

There were minimal differences for gain to feed ratio (G:F) among treatments (Table 1). Pigs fed MT were different from CON only during days 21–28 and over the entire trial. Pigs fed MT-YC were not different from MT throughout the trial. The only observed difference between MT-YF and MT was during days 0–7, where MT-YF tended to have increased (*p* = 0.086) G:F.

### 2.2. Immunological, Oxidative, Hematological, and Biochemical Analysis

Concentrations of the immune parameter IgG were not different among pigs (Table 2). The concentration of TNFα tended to be reduced (*p* = 0.060) in pigs fed MT-YF compared with MT. Analysis of MDA showed that treatments did not differ for both serum and jejunum tissue lipid peroxidation (Table 2). The level of 8-OHdG was increased (*p* < 0.05) in pigs fed MT in contrast to pigs fed CON, whereas pigs fed MT-YF tended to have a reduction (*p* = 0.067) in 8-OHdG compared with MT (Table 2). 

**Table 2:** Immunological and oxidative stress parameters as measured in the serum and intestinal jejunum tissue after pigs consumed 4.8 mg/kg DON and 0.3 mg/kg ZEA with or without yeast based feed additives.

Item	Treatment ^1^				SEM	Contrast *p* value: MT *vs.*		
	CON	MT	MT-YC	MT-YF		CON	MT-YC	MT-YF
Immunological parameters ^2^								
IgG, mg/mL	13.6	15.1	15.1	13.0	1.6	0.502	0.998	0.359
TNFα, pg/mL	88.0	108.3	97.2	82.0	9.6	0.171	0.412	0.060
Oxidative stress parameters ^2^								
MDA, µM	14.7	10.2	11.7	12.2	2.2	0.148	0.632	0.511
8-OHdG, ng/mL	0.71	1.49	1.45	0.77	0.27	0.048	0.917	0.067
Jejunum MDA, µM/mg protein	1.44	1.13	1.39	1.44	0.25	0.385	0.467	0.384

^1^ CON: control without significant DON or ZEA; MT: 4.8 mg/kg DON and 0.3 mg/kg ZEA. Composition of diets MT-YC: MT + 2 g/kg of a yeast cell wall product (Integral, Alltech Inc., Nicholasville, KY, USA); and MT-YF: MT + 2 g/kg of a yeast fermentation product (Original XPC, Diamond V, Cedar Rapids, IA, USA); ^2^ IgG: immunoglobulin G; TNFa: tumor necrosis factor alpha; MDA: malondialdehyde; 8-OHdG: 8-hydroxy-deoxyguanosine.

The CBC analysis on day 42 showed few differences among treatments (Table 3). Pigs fed MT tended to have increased MCH (*p* = 0.087) and MCV (*p* = 0.050), and had decreased platelet levels (*p* < 0.05) in contrast to pigs fed CON. These pigs also had a reduced RBC count (*p* < 0.05) from pigs fed CON, and at the same time pigs fed MT-YC had increased (*p* ≤ 0.05) RBC count and MT-YF tended to have an increased (*p* = 0.064) RBC count. Serum biochemical parameter analysis showed few effects of mycotoxins or additives (Table 4). Pigs fed MT-YC had increased (*p* < 0.05) ALT compared with MT, whereas pigs fed MT-YF tended to have increased (*p* = 0.057) AST than MT. Serum calcium, cholesterol, and protein levels were decreased (*p* < 0.05) in pigs fed MT in contrast to pigs fed CON. 

**Table 3:** Hematological analysis for pigs consuming 4.8 mg/kg DON and 0.3 mg/kg ZEA with or without yeast based feed additives.

Item	Treatment ^1^				SEM	Contrast *p* value: MT *vs.*		
	CON	MT	MT-YC	MT-YF		CON	MT-YC	MT-YF
Hematocrit,%	35.8	34.7	36.7	35.9	0.9	0.385	0.107	0.337
Hemoglobin, g/dL	11.0	10.6	11.2	11.0	0.3	0.343	0.103	0.270
MCH ^2^, pg	16.9	17.6	17.5	17.2	0.3	0.087	0.764	0.338
MCHC ^2^, g/dL	30.7	30.7	30.7	30.8	0.3	0.941	0.911	0.824
MCV ^2^, fL	55.0	57.6	57.0	56.1	0.9	0.050	0.651	0.263
Platelet, 10^3^/µL	520.7	355.7	459.3	344.6	52.7	0.047	0.202	0.889
RBC ^2^, 10^6^/µL	6.51	6.04	6.43	6.40	0.13	0.019	0.049	0.064
WBC ^2^, 10^3^/µL	17.6	18.7	17.7	17.3	1.0	0.450	0.462	0.324
Basophil, 10^3^/µL	0.12	0.13	0.18	0.15	0.03	0.842	0.237	0.635
Eosinophil, 10^3^/µL	0.43	0.38	0.36	0.46	0.10	0.704	0.882	0.551
Lymphocyte, 10^3^/µL	10.4	11.0	10.4	11.0	0.7	0.522	0.508	0.939
Monocyte, 10^3^/µL	0.70	0.93	0.88	0.74	0.09	0.105	0.712	0.170
Neutrophil, 10^3^/µL	5.99	6.21	5.88	5.00	0.73	0.832	0.749	0.244

^1^ CON: control without significant DON or ZEA; MT: 4.8 mg/kg DON and 0.3 mg/kg ZEA. Composition of diets MT-YC: MT + 2 g/kg of a yeast cell wall product (Integral, Alltech Inc., Nicholasville, KY, USA); and MT-YF: MT + 2 g/kg of a yeast fermentation product (Original XPC, Diamond V, Cedar Rapids, IA, USA); ^2^ MCH: mean corpuscular hemoglobin; MCHC: mean corpuscular hemoglobin concentration; MCV: mean corpuscular volume; RBC: red blood cell count; WBC: white blood cell count.

**Table 4:** Serum biochemical analysis for pigs consuming 4.8 mg/kg DON and 0.3 mg/kg ZEA with or without yeast based feed additives.

Item	Treatment ^1^				SEM	Contrast *p* value: MT *vs.*		
	CON	MT	MT-YC	MT-YF		CON	MT-YC	MT-YF
Albumin, g/dL	2.51	2.21	2.23	2.46	0.14	0.140	0.943	0.229
Albu:globulin ^2^	1.11	1.06	1.01	1.26	0.13	0.750	0.811	0.270
Alk phos ^2^, U/L	201	174	210	165	16	0.250	0.133	0.701
ALT ^2^, U/L	37.6	35.9	46.1	42.1	2.9	0.670	0.016	0.127
AST ^2^, U/L	32.0	37.0	43.1	51.1	5.1	0.487	0.394	0.057
Bilirubin, mg/dL	0.10	0.10	0.10	0.10	-	-	-	-
BUN:creatinine ^2^	24.4	21.9	21.3	21.3	1.3	0.166	0.753	0.753
Ca, mg/dL	9.89	9.27	9.30	9.36	0.14	0.005	0.888	0.672
Cl, mEq/L	105	106	106	104	1	0.560	1.000	0.137
Cholesterol, mg/dL	89.1	69.7	75.6	67.1	6.6	0.044	0.528	0.781
CPK ^2^, U/L	444	974	691	1,768	561	0.502	0.719	0.317
Creatinine, mg/dL	0.80	0.80	0.80	0.78	0.04	1.000	1.000	0.492
Globulin, g/dL	2.37	2.17	2.26	2.17	0.18	0.425	0.731	1.000
Glucose, mg/dL	88.0	82.6	81.4	85.0	3.9	0.352	0.843	0.675
Na:K ^2^	22.4	23.0	23.7	24.3	0.7	0.579	0.489	0.218
P, mg/dL	10.6	10.4	10.5	10.7	0.3	0.513	0.616	0.399
K, mEq/L	6.59	6.31	6.21	6.04	0.19	0.343	0.725	0.343
Protein, g/dL	4.89	4.39	4.49	4.63	0.13	0.014	0.600	0.210
Na, mEq/L	146	145	147	145	1	0.711	0.408	0.926
Urea N, mg/dL	19.3	17.1	17.0	16.0	1.1	0.184	0.928	0.473

^1^ CON: control without significant DON or ZEA; MT: 4.8 mg/kg DON and 0.3 mg/kg ZEA. Composition of diets MT-YC: MT + 2 g/kg of a yeast cell wall product (Integral, Alltech Inc., Nicholasville, KY, USA); and MT-YF: MT + 2 g/kg of a yeast fermentation product (Original XPC, Diamond V, Cedar Rapids, IA, USA); ^2^ Albu:globulin: albumin to globulin ratio; Alk phos: alkaline phosphatase; ALT: alanine aminotransferase; AST: aspartate aminotransferase; BUN:creatinine: BUN to creatinine ratio: CPK: creatine phosphokinase; Na:K: sodium to potassium ratio.

**Table 5:** Organ morphology of pigs consuming 4.8 mg/kg DON and 0.3 mg/kg ZEA with or without yeast based feed additives.

Item	Treatment ^1^				SEM	Contrast *p* value: MT *vs.*		
	CON	MT	MT-YC	MT-YF		CON	MT-YC	MT-YF
Weights, g								
Liver	716	603	600	676	41	0.094	0.961	0.271
Kidney	63.7	51.8	56.0	59.4	5.3	0.133	0.582	0.330
Spleen	44.9	36.5	44.5	43.2	3.5	0.112	0.130	0.204
Uterus	23.3	27.6	21.7	26.9	3.6	0.418	0.312	0.910
Wt as% BW								
Liver	1.26	1.26	1.21	1.29	0.04	0.966	0.365	0.605
Kidney	0.11	0.11	0.11	0.11	0.00	0.648	0.608	0.486
Spleen	0.08	0.08	0.09	0.08	0.00	0.783	0.042	0.446
Uterus	0.04	0.06	0.04	0.05	0.01	0.063	0.175	0.457
Jejunum, µm								
Villi height	484	441	466	468	22	0.199	0.450	0.413
Crypt depth	318	331	311	318	13	0.513	0.326	0.499
Uterus, µm								
Longitudinal muscle	219	221	249	240	29	0.965	0.464	0.641
Circular muscle	451	401	555	458	49	0.457	0.037	0.398
Submucosa	1687	1620	1660	1704	112	0.694	0.915	0.625
Mucosa	31.5	31.2	32.1	31.3	2.8	0.938	0.826	0.976
Vulva								
Width, mm	22.1	24.0	24.3	26.1	1.0	0.182	0.834	0.126
Height, mm	27.7	29.6	30.1	31.1	1.2	0.263	0.728	0.342

^1^ CON: control without significant DON or ZEA; MT: 4.8 mg/kg DON and 0.3 mg/kg ZEA. Composition of diets MT-YC: MT + 2 g/kg of a yeast cell wall product (Integral, Alltech Inc., Nicholasville, KY, USA) and MT-YF: MT + 2 g/kg of a yeast fermentation product (Original XPC, Diamond V, Cedar Rapids, IA, USA).

### 2.3. Internal Organ Health

Liver weight tended (*p* = 0.094) to be reduced in pigs fed MT compared with CON (Table 5). Spleen weight as a percent of BW was increased (*p* < 0.05) in pigs fed MT-YC in contrast to MT, whereas uterus weight as a percent of BW tended (*p* = 0.063) to be increased in pigs fed MT compared with CON. Uterine tissue morphology did not differ among treatments except for the circular muscle thickness, which was thicker (*p* < 0.05) in pigs fed MT-YC compared with MT. Liver damage was minimal for all pigs (Table 6). However, pigs fed MT had increased (*p* < 0.05) hepatic hydropic degeneration compared with pigs fed CON, whereas MT-YC tended (*p* = 0.079) to reduce this damage and MT-YF reduced (*p* < 0.05) the damage compared to MT. All other forms of liver and kidney damages were not observed. 

**Table 6:** Tissue damage of pigs consuming 4.8 mg/kg DON and 0.3 mg/kg ZEA with or without yeast based feed additives.

Item	Treatment ^1^				SEM	Contrast *P* value: MT *vs.*		
	CON	MT	MT-YC	MT-YF		CON	MT-YC	MT-YF
Liver ^2^								
Hydropic degeneration	1.14	1.71	1.29	1.14	0.17	0.022	0.079	0.022
Vacuolation	1.00	1.00	1.00	1.00	0.00	-	-	-
Necrosis	1.00	1.00	1.00	1.00	0.00	-	-	-
Inflammation	1.00	1.00	1.00	1.00	0.00	-	-	-
Karyomegaly	1.00	1.43	1.29	1.57	0.26	0.245	0.695	0.695
Fibrosis	1.29	1.14	1.29	1.57	0.18	0.579	0.579	0.105
Bile ductile hyperplasia	1.14	1.29	1.43	1.14	0.17	0.558	0.558	0.558
Kidney ^2^								
Vacuolation	1.00	1.00	1.00	1.00	0.00	-	-	
Necrosis	1.00	1.00	1.00	1.00	0.00	-	-	-
Inflammation	1.57	1.43	1.29	1.71	0.19	0.606	0.606	0.307
Regeneration	1.00	1.00	1.00	1.00	0.00	-	-	-
Protein casts	1.00	1.00	1.00	1.00	0.00	-	-	-
Fibrosis	1.00	1.00	1.00	1.00	0.00	-	-	-

^1^ CON: control without significant DON or ZEA; MT: 4.8 mg/kg DON and 0.3 mg/kg ZEA. Composition of diets MT-YC: MT + 2 g/kg of a yeast cell wall product (Integral, Alltech Inc., Nicholasville, KY, USA); and MT-YF: MT + 2 g/kg of a yeast fermentation product (Original XPC, Diamond V, Cedar Rapids, IA, USA); ^2^ Liver and kidney microscopic examinations indicating the percent of damage to tissue: normal to minimal (0%–5%); mild (5%–15%); moderate (15%–40%); severe (greater than 40%).

## 3. Discussion

### 3.1. Impact of Mycotoxins on Pigs

The current study shows that mycotoxins from naturally contaminated corn had a strong impact on pig performance and health. Deoxynivalenol and ZEA are harmful mycotoxins for swine, reducing both growth and reproductive performance [4,5]. Vomiting is characteristic of high DON consumption and is shown at concentrations of 4.4–20 mg/kg [2,14]. In our current study, no vomiting was observed. However, the consumption of 4.8 mg/kg DON and 0.3 mg/kg ZEA by pigs altered growth performance. Over the entire trial period, the ADG of pigs fed the mycotoxin contaminated diet was decreased by 33% compared with CON. Other research shows a similar trend for reduced ADG of pigs consuming both low and high concentrations of 0.2–11 mg/kg DON with 0.3–2 mg/kg ZEA [5,15,16,17,18]. Data from Dersjant-Li *et al.* [19] showed that feeding DON as low as 0.6 mg/kg can cause a 5% reduction in growth of pigs. Based on this information from Dersjant-Li *et al.* [19], calculations predict that 4.8 mg/kg feed DON should cause a 40% reduction in ADG. The reduction in ADG in our current study came close to this prediction at a 33% decrease. 

The effects of DON on feed intake can be variable. Deoxynivalenol is not shown to decrease ADFI at low concentrations of 0.28 mg/kg [20]. However, Williams *et al.* [2] showed a decrease in ADFI after feeding high concentrations of 4.4–11 mg/kg DON with 0.5–2.0 mg/kg ZEA. In our current study, the consumption of DON and ZEA reduced feed intake by 38% in contrast to pigs fed CON. With these results, it is apparent that the combination of DON and ZEA in our trial had a greater effect on pig feed intake than gain. Previous research has speculated that the strong impact of DON on feed intake is a result of changes in brain neurotransmitter concentrations [18]. Work by Shen *et al.* [18] shows that consumption of DON can lead to an increase in serotonin levels. Serotonin can increase lethargy and lead to lower feed intake.

To further understand how the mycotoxins DON and ZEA impact pigs, this study investigated the effects on immunity, oxidative stress, and organ health. Unexpectedly, the addition of 4.8 mg/kg DON and 0.3 mg/kg ZEA to the diets did not greatly alter the internal systems of the pigs. However, there were minor effects which together may increase the overall challenge to the animal. 

Immunoglobulin G (IgG), the pro-inflammatory cytokine tumor necrosis factor alpha (TNFα), and blood cell levels were analyzed as indicators of mycotoxin challenge to the immune system. Although no effect was observed for the immune parameters IgG and TNFα between MT and CON in this trial, previous research shows varying effects of mycotoxins on these immune parameters. In one case, Accensi *et al.* [20] found that low levels of DON up to 0.90 mg/kg did not affect IgG concentrations, whereas Shen *et al.* [18] indicated that 3 mg/kg DON caused a decrease in IgG. As a result of this varying data, it may be the case that other factors play a role in immune response, such as previous health status, disease challenges, gut quality, or even farm management.

Another indicator of immune system function is total WBC count or the WBC subsets neutrophils, monocytes, lymphocytes, basophils, and eosinophils. These immune parameters were not altered by DON and ZEA in this study. However, Pinton *et al.* [21] showed that monocyte numbers can increase in pigs fed 2.2–2.5 mg/kg DON. Platelet and RBC counts can also be an indicator of pig health. In the current study, feeding of contaminated feed did appear to significantly alter these blood measurements indicating that the mycotoxins may have a slight effect on some blood characteristics. However, other studies have not shown an effect of DON and ZEA on these hematological parameters [20]. Despite the impacts on these blood components, pigs naturally have a wide range of normal levels and thus these values do not fall outside of the suggested reasonable range [22,23]. The oxidative damage parameters MDA and 8-OHdG were used as indicators of lipid peroxidation and DNA damage, respectively. Lipid peroxidation was not affected by consumption of these mycotoxins, as measured in both the serum and jejunum tissue. Few studies have determined the ability of DON and ZEA to cause oxidative stress, especially in pigs. However, Borutova *et al.* [24] showed that broilers fed 3.4–8.2 mg/kg DON and 3.4–8.3 mg/kg ZEA had increased MDA in liver tissue but did not affect duodenum mucosa MDA. In contrast, Chaytor *et al.* [25] indicated that a combination of 0.182 mg/kg AFB1 with 0.768 mg/kg DON caused lipid peroxidation. Although lipid peroxidation was not observed in our current study, it appears that DON and ZEA may have a potential to cause lipid peroxidation. Whether this damage occurs or not may be a result of the concentration of these mycotoxins, other mycotoxins they are combined with, or other stress or disease challenges the pig is facing. Previous studies have shown a similar effect of DON on DNA damage, where 4–10 mg/kg DON resulted in DNA damage in chickens and young pigs [26,27]. 

Mycotoxins may also impact internal organ health. One method of measuring organ health is through serum biochemical analysis. Although few differences were observed, pigs fed the mycotoxin contaminated feed did have lower serum calcium, cholesterol, and protein concentrations compared with pigs fed the control. Researchers from Bergsjø *et al.* [28] observed similar effects on serum biochemistry in pigs, where DON reduced serum calcium, phosphorus, albumin, and total protein. This impact of DON on these biochemical parameters may be simply due to the fact that the pigs fed the mycotoxins had reduced feed intake which may result in decreased nutrient intake and in turn a reduction of protein and mineral excretion from the liver. Simultaneously, due to the fact that DON may inhibit protein synthesis, this may also be a cause for decreased protein levels [28]. 

The internal organ weights of pigs, including the liver, kidney, spleen, uterus, and jejunum were minimally affected by mycotoxin contamination. Uterus weight as a percent of BW did tend to be increased due to consumption of the mycotoxins. Other research has shown that uterus weight as a percent of BW can be increased in pre-pubertal gilts after consuming 3.9 mg/kg DON and 0.42 mg/kg ZEA, while other research has shown uterus weight to increase only after mating [29,30]. Previous research has also documented that high concentrations of 1–5 mg/kg ZEA can cause vulva swelling in gilts [31]. In our current study, all gilts were pre-pubertal (less than six months of age) which may be why ZEA did not have a strong effect on their reproductive tract.

Histological evaluation of tissue samples was completed in this trial to investigate the occurrence of organ damage. It has been previously documented that 0.9–1 mg/kg DON can cause organ damage including fibrosis, necrosis, blood vessel thickening, and hemorrhage [4,5,16]. In our current study, the ingested mycotoxins caused minimal tissue damage. Although the damage was low (less than 5% damage), hydropic degeneration was observed in the liver of pigs fed MT. 

Together, the results for this trial indicate that mycotoxins do not only impact pig performance, but can also cause immunological, oxidative stress, and organ health damages. As a result of these effects of mycotoxins on pigs, it is crucial that mycotoxin mitigation strategies be included in the ration to assist pigs in coping with mycotoxin challenges. 

### 3.2. Effects of Mycotoxin Mitigation Strategies

The feeding of the yeast products provided varied degrees of benefits for pigs when challenged with DON and ZEA. The additive in MT-YCW (Integral, Alltech Inc., Nicholasville, KY, USA) contains hydrolyzed yeast, which includes the cell wall fraction of the yeast. The cell wall is primarily composed of polysaccharides such as β-glucan, which is the inner layer that makes up 50%–60% of the cell wall dry weight [10]. The β-glucan of yeast can from single or triple helix polysaccharide chains which can give adsorptive capacities for mycotoxins through hydrogen and ionic bonding, and van der Waals interactions [11]. Previous *in vitro* studies have shown that β-glucan isolated from yeast cell walls has the capacity to adsorb up to 50% of ZEA [32,33]. Yeast β-glucan has also been shown *in vitro* to adsorb aflatoxin B1 (AFB1), DON, ochratoxin A, and patulin. 

The additive in MT-YF (Original XPC, Diamond V, Cedar Rapid, IA, USA) is the dried anaerobic fermentation product from *Saccharomyces cerevisiae* and contains not only the β-glucan cell wall fraction but also the outer wall layer of mannoproteins and fermentation metabolites [13,34]. This yeast fermentation product has been shown to provide several beneficial prebiotic effects, such as improving pig growth performance, modulating the immune system, and improving gut morphology [12,13]. The yeast fermentation product may also play a role in adsorbing mycotoxins, due to the fact that it contains β-glucan. Together, these effects of the yeast fermentation product may improve the ability of the animal to fight mycotoxins. 

When looking at the efficacy of the two mycotoxin mitigation strategies, pigs fed these yeast additives had varied responses to the mycotoxin challenge. Pigs fed MT-YC with the yeast cell wall product were not different from MT for all measurements of BW, ADG, ADFI, and G:F. Pigs receiving the yeast fermentation product in MT-YF had an inconsistent ability to improve performance on an individual week, but over the entire trial period MT-YF improved ADG by 19% when added to the mycotoxin contaminated diet and tended to improve ADFI by 17%. Few studies have determined the ability of yeast based additives to reduce these mycotoxin effects in pigs. However, Osweiler *et al.* [35] showed that in broilers, that adding 0.125% of a yeast fermentation product had the benefit of improving growth performance of birds when added to a diet with the mycotoxin AFB1 at 2.28 mg/kg. 

The two mycotoxin mitigation strategies had some benefits for reducing the effects of mycotoxins on the internal organ systems of pigs. Damage to DNA was the only form of oxidative stress impacted by consumption of the mycotoxins. This oxidative stress parameter tended to be lower in pigs fed MT-YF. On the other hand, the additive MT-YC did not alter the levels of oxidative DNA damage observed when the mycotoxins were fed. Few studies have determined the ability of yeast additives to reduce oxidative stress. Thus, the current study provides important information on the ability of yeast additives to reduce the oxidative stress when pigs are challenged by mycotoxins. 

When considering organ health, serum liver biochemistry showed that the mycotoxins DON and ZEA lowered serum calcium, cholesterol, and protein concentrations. However, the biochemical parameters of pigs fed either yeast additive remained similar to the mycotoxin fed pigs. Additionally, the hepatic enzymes ALT and AST were altered by treatment with the mitigation strategies regardless of mycotoxin contamination. Here, pigs fed MT-YC had increased ALT compared with MT, whereas pigs fed MT-YF tended to have increased AST compared with MT. Both ALT and AST are enzymes involved in amino acid and protein metabolism [36]. If hepatic injury occurs, ALT may be released, while AST can be released due to liver, heart, or muscle damage [37]. Although levels of ALT and AST showed an increase due to the inclusion of the yeast additives, these increases were trivial and do not necessarily indicate liver or muscle damage. Based on values determined by Friendship *et al.* [22] and Odink *et al.* [38], healthy pigs have wide ranges of serum ALT and AST levels where all treatments in the current study did not fall outside of the normal range of 8–46 U/L ALT and 16–94 U/L AST. 

The internal organ weights of pigs, including the liver, kidney, spleen, uterus, and jejunum were minimally affected by mycotoxin contamination or the addition of the yeast additives. Although the damage was low (less than 5% damage), hydropic degeneration was observed in the liver of pigs fed MT tending to have increased damage compared to pigs fed CON, MT-YC, and MT-YF. Previous research by Weaver *et al.* [39] documented that feed additives containing dried yeast and yeast fermentation materials may be beneficial in reducing organ damages in pigs caused by feeding 0.15 mg/kg AF with 1.1 mg/kg DON. 

## 4. Experimental Section 

### 4.1. Animals and Experimental Diets

Eighty four gilts (9.1 ± 0.1 kg, crossbred pigs, Smithfield Premium Genetics, Rose Hill, NC, USA) averaged six weeks of age, were used in this study. Pigs were housed in solid concrete floor indoor pens (1.42 × 3.86 m) at the North Carolina State University Swine Evaluation Station (Clayton, NC, USA). Pigs were grouped by body weight (BW) and randomly assigned to four treatments within a BW group. Each treatment had seven replicates and three pigs per pen. 

Corn naturally contaminated with mycotoxins was identified and the mycotoxin concentrations were confirmed by Veterinary Diagnostic Laboratory at North Dakota State University (Fargo, ND, USA). Corn was analyzed for 19 mycotoxins including DON and ZEA. Quantification of DON and ZEA was conducted using GC-MS. Corn contained DON (25 mg/kg) and ZEA (3.4 mg/kg) and used to make experimental diets (Table 7). This contaminated corn was blended with corn without mycotoxins in order to reach analyzed levels of 4.8 mg/kg DON and 0.3 mg/kg ZEA in the final diets. Non-contaminated corn was also used to formulate a control without mycotoxins. Mycotoxin analysis in corn and final diets was completed by collecting 10 samples from different locations to obtain a representative mixture [40,41,42]. The 10 samples were combined and thoroughly blended together before two subsamples were collected for analysis of mycotoxin content. Mycotoxin contaminants were measured by the North Dakota State Veterinary Diagnostic Laboratory (Fargo, ND, USA). The level of DON in contaminated grains was determined using gas chromatography-mass spectrometry with a quantitation limit of 500 μg/kg. 

Pigs were fed experimental diets based on their assigned treatment groups representing: CON (control); MT (4.8 mg DON/kg and 0.3 mg ZEA/kg); MT-YC (MT + 2 g/kg of a yeast cell wall product, Integral, Alltech Inc., Nicholasville, KY, USA); and MT-YF (MT + 2 g/kg of a yeast fermentation product, Original XPC, Diamond V, Cedar Rapids, IA, USA). The yeast cell wall based product in MT-YC is composed of hydrolyzed yeast which includes the cell wall fraction of the organism, whereas the yeast fermentation product in MT-YF is the dried anaerobic fermentation product from *Saccharomyces cerevisiae*. All experimental diets were fed for 42 days, and average daily gain (ADG), average daily feed intake (ADFI), and gain to feed ratio (G:F) were determined. During the entire experimental period, all pigs had free access to feed and water. Concentrations of essential nutrients met requirements suggested by the National Research Council (1998). A protocol for the use of animals in this study was approved by North Carolina State University Animal Care and Use Committee.

**Table 7:** Composition of experimental diets (as-fed basis).

Ingredient	Treatment ^1^	
	CON (%)	MT (%)
Ground yellow corn ^2^	72.00	52.00
Ground yellow corn with mycotoxins ^3^	0.00	20.00
Soybean meal, dehulled	25.30	25.30
Salt	0.30	0.30
Vitamin premix ^4^	0.03	0.03
Trace mineral premix ^5^	0.15	0.15
Dicalcium phosphate	0.90	0.90
Ground limestone	0.70	0.70
Poultry fat	0.62	0.62
Calculated composition		
Dry matter, %	89.6	89.6
Metabolizable energy, Mcal/kg	3.37	3.37
Crude protein, %	18.0	18.0
True ileal idgestible Lys, %	0.83	0.83
True ileal idgestible Cys + Met, %	0.54	0.54
True ileal idgestible Trp, %	0.18	0.18
True ileal idgestible Thr, %	0.58	0.58
Ca, %	0.61	0.61
Available P, %	0.23	0.23
Total P, %	0.54	0.54
Deoxynivalenol (DON), mg/kg	0.00	5.00
Zearalenone (ZEA), mg/kg	0.00	0.68
Analyzed composition		
DM, %	87.7	88.1
CP, %	16.5	16.1
Deoxynivalenol ^6^, mg/kg	0.36	4.82
Zearalenone ^6^, mg/kg	ND ^7^	0.33

^1^ CON: control without significant DON or ZEA; MT: 4.8 mg/kg DON and 0.3 mg/kg ZEA. Composition of diets MT-YC: MT + 2 g/kg of a yeast cell wall product (Integral, Alltech Inc., Nicholasville, KY, USA); and MT-YF: MT + 2 g/kg of a yeast fermentation product (Original XPC, Diamond V, Cedar Rapids, IA, USA). MT-YC and MT-YF have identical composition to MT except for the addition of the feed additive; ^2^ Corn contained 0.23 mg/kg DON, other mycotoxins not detectable; ^3^ Corn contained 25 mg/kg DON, 4 mg/kg 15-acetyl deoxynivalenol, 3.4 mg/kg ZEA, <2 mg/kg fumonisin, and <0.02 mg/kg aflatoxin. Analysis of mycotoxins in corn was completed by North Dakota State University Veterinary Diagnostic Laboratory (Fargo, ND, USA) by HPLC; ^4^ The vitamin premix provided the following per kilogram of complete diet: 6613.8 IU of vitamin A as vitamin A acetate; 992.0 IU of vitamin D_3_; 19.8 IU of vitamin E; 2.64 mg of vitamin K as menadione sodium bisulfate; 0.03 mg of vitamin B_12_; 4.63 mg of riboflavin; 18.52 mg of D-pantothenic acid as calcium panthonate; 24.96 mg of niacin; 0.07 mg of biotin; ^5^ The trace mineral premix provided the following per kilogram of complete diet: 4.0 mg of Mn as manganous oxide; 165 mg of Fe as ferrous sulfate; 165 mg of Zn as zinc sulfate; 16.5 mg of Cu as copper sulfate; 0.30 mg of I as ethylenediamine dihydroiodide; and 0.30 mg of Se as sodium selenite; ^6^ Dietary DON and ZEA concentration based on averages obtained from analysis by ELISA assay (AgraQuant Deoxynivalenol or AgraQuant Zearalenone, Romer Labs, Union, MO, USA); ^7^ Zearalenone concentration in CON was not detectable as the value was below the limit of detection, 0.20 mg/kg, for this ELISA assay.

### 4.2. Blood Sampling 

The pig with the median initial BW from each pen was bled on day 42 for immunological, hematological, and biochemical analysis. Blood was collected in Monovette tubes (Sarstedt, Newton, NC, USA) without anticoagulant to obtain serum for liver biochemistry, immunoglobulin, cytokine, and oxidative stress parameters. Blood was allowed to clot before centrifuging for 15 min at 3000 *g* (4 °C) to collect serum, and samples were stored at −80 °C until analyzed. Blood samples were also collected in tubes containing EDTA to obtain whole blood for hematological analysis. 

### 4.3. Immunological and Oxidative Stress Parameters 

The immunoglobulin subset immunoglobulin G (IgG) was measured via enzyme linked immunosorbent assay (ELISA), as described by the manufacturer (Bethyl, Montgomery, TX, USA). Goat anti-pig IgG was used as a capture antibody to coat wells. Serum samples were diluted to 1:100,000. Horseradish peroxidase goat anti-pig IgG was used as a detection antibody in combination with the tetramethylbenzidine enzyme substrate. A stop solution of 0.18 M sulfuric acid (H_2_SO_4_) was used to stop the enzyme-substrate reaction. Absorbance was read at 450 nm using a Synergy HT ELISA plate reader (BioTek Instruments, Inc., Winooski, VT, USA) and Gen5 data analysis software (BioTek Instruments, INC, Winooski, VT, USA). Samples were quantified relative to the standard curve constructed with known amounts of pig IgG. The ELISA IgG detection limit was 7.8–500 ng/mL.

The cytokine tumor necrosis factor alpha (TNFα) was measured in serum by ELISA following the manufactures procedure (R&D Systems, Minneapolis, MN, USA). A total of 50 μL assay dilute was added to microplate wells coated with a monoclonal antibody specific to porcine TNFα, followed by 50 μL of standard, control, or sample. Detection occurred by the use of a color reagent substrate and a stop solution of diluted hydrochloric acid, and absorbance was read at 450 nm and 540 nm. The detection limit range for TNFα was 2.8–5.0 pg/mL.

The parameters malondialdehyde (MDA) and 8-hydroxy-deoxyguanosine (8-OHdG) were measured in serum as indicators of oxidative stress. Lipid peroxidation was measured by MDA using TBARS assay following the manufactures protocol (Cell Biolabs, Inc., San Diego, CA, USA). Samples and standards were added to microcentrifuge tubes, followed by SDS lysis solution and thiobarbituric acid (TBA). All tubes were incubated at 95 °C for 50 min, and then placed on ice for 5 min to cool before being centrifuged at 3000 rpm for 15 min. The supernatant was removed to a 96 well microplate and absorbance was read at 532 nm using the Synergy HT ELISA plate reader. The MDA content was determined in samples by comparison with the MDA standard curve. 

Production of 8-OHdG was determined by ELISA (Cell Biolabs, Inc., San Diego, CA, USA) following protocol to determine oxidative DNA damage. Undiluted samples were added to an 8-OHdG conjugate coated microplate, followed by diluted anti-8-OHdG antibody, and finally diluted secondary antibody enzyme conjugate. After incubation, the provided stop solution was added to each well, and allowed to incubate for 8–10 min before being stopped with a stop solution in order to achieve a color change which was not over saturated. Samples were then measured at 450 nm and concentration determined based on the standard curve.

### 4.4. Hematological and Biochemical Assays 

Whole blood with EDTA was sent to Antech Diagnostics (Cary, NC, USA) for complete blood counting (CBC). Measurements included hematocrit, hemoglobin, mean corpuscular hemoglobin (MCH), mean corpuscular hemoglobin concentration (MCHC), mean corpuscular volume (MCV), platelet number, red blood cell (RBC) count, white blood cell (WBC) count, basophils, eosinophils, lymphocytes, monocytes, and neutrophils. 

Concentrations of serum alanine aminotransferase (ALT), albumin, alkaline phosphatase, aspartate aminotransferase (AST), bilirubin, BUN to creatinine ratio (BUN:creatinine), calcium, chloride, cholesterol, creatinine, creatine phosphokinase (CPK), globulin, glucose, phosphorus, potassium, sodium, and urea nitrogen were measured (Antech Diagnostics, Cary, NC, USA) for determination of liver biochemistry.

### 4.5. Organ Collection and Analysis

Measurements of vulva height and width were taken from one pig per pen (median initial BW pig) on day 42, before being anesthetized for tissue collection. Samples of the liver, left kidney, spleen, jejunum, and uterus were collected and weighed before being fixed in either 10% buffered formalin or liquid nitrogen. Tissues in formalin were sent to the North Carolina State University Histopathology Laboratory (College of Veterinary Medicine, Raleigh, NC, USA) for hematoxylin and eosin (H&E) staining and slide preparation. Samples in liquid nitrogen were stored in at −80 °C until further analysis.

Microscopic examination of tissue damage for the liver and kidney were measured by a histopathologist blinded to treatment (College of Veterinary Medicine, Raleigh, NC, USA). Damages were based on the degree of change observed with values of 1: normal to minimal damage (0%–5%); 2: mild (5%–15%); 3: moderate (15%–40%); 4: severe (higher than 40%). Liver damage measurement included bile ductule hyperplasia, fibrosis, hydropic degeneration, inflammation, karyomegaly, necrosis, and vacuolation. Kidney damage measurement included fibrosis, inflammation, necrosis, protein casts, regeneration, and vacuolation. Jejunum villi length and crypt depths were measured using an Olympus Vanox microscope (Olympus Corporation, Center Valley, PA, USA) and Spot Advanced software program (SPOT Imaging Solutions, Sterling Heights, MI, USA). Jejunum tissue was also measured for MDA concentration. This analysis was completed by homogenizing tissue in PBS and resulting supernatant was analyzed for MDA by TBARS assay (Cell Biolabs, INC., San Diego, CA, USA) as previously described. Tissue samples were also analyzed for protein content (BCA Protein Assay Kit, Pierce Biotechnology, Rockford, IL, USA) after a 1:10 dilution for determination of MDA per mg protein. Measurements of uterus longitudinal muscle, circular muscle, submucosa, and mucosa thicknesses were collected using the Olympus Vanox microscope and Spot Advanced software. 

### 4.6. Statistical Analysis 

Data was analyzed using the GLM procedures of SAS (SAS Inst. Inc., Cary, NC, USA) following a completely randomized block design with pigs blocked by initial BW. A pen was considered as the experimental unit. Separation of means was completed using the PDIFF option of SAS. Probability values less than 0.05 were considered statistically significant and between 0.05 and 0.10 as trends.

## 5. Conclusions

Collectively, the results of this study indicate that the consumption of 4.8 mg/kg DON and 0.3 mg/kg ZEA reduced ADG by 33% and ADFI by 38%. The stronger effect of these mycotoxins on feed intake may have been the primary reason for the decrease in growth performance. However, some slight impacts on organ health, oxidative stress, and immunity were observed as well which may have played a further role in the reduction of pig performance. 

The addition of the two mycotoxin mitigation strategies resulted in varying benefits to pigs. The yeast fermentation additive did provide some ability to improve the performance of pigs that were simultaneously consuming 4.8 mg/kg DON and 0.3 mg/kg ZEA. This additive also reduced some of the effects of mycotoxins on the pig health. While the yeast cell wall product did not significantly improve the growth performance of pigs consuming mycotoxins, this feed additive did reduce some of the internal damage associated with consuming the mycotoxins. Generally, the addition of both the yeast cell wall product and yeast fermentation product showed some benefits for reducing the effects of mycotoxins on pigs. These responses may vary under different mycotoxin concentrations, types, and mixtures.
